# Essential oil from halophyte *Lobularia maritima*: protective effects against CCl_4_-induced hepatic oxidative damage in rats and inhibition of the production of proinflammatory gene expression by lipopolysaccharide-stimulated RAW 264.7 macrophages

**DOI:** 10.1039/c9ra05885k

**Published:** 2019-11-11

**Authors:** Anis Ben Hsouna, Sabah Dhibi, Wissal Dhifi, Rania Ben Saad, Faical Brini, Najla Hfaidh, Wissem Mnif

**Affiliations:** Department of Life Sciences, Faculty of Sciences of Gafsa Zarroug 2112 Gafsa Tunisia; Laboratory of Biotechnology and Plant Improvement, Centre of Biotechnology of Sfax Tunisia; Unit of Macromolecular Biochemistry and Genetics, Faculty of Sciences of Gafsa Sidi Ahmed Zarrouk, 2112 Gafsa Tunisia sabahdhibi7@gmail.com; LR17-ES03 Physiopathology, Food and Biomolecules, Higher Institute of Biotechnology of Sidi Thabet, Biotechpole Sidi Thabet 2020 Ariana Tunisia; Department of Chemistry, Faculty of Sciences and Arts in Balgarn, University of Bisha Bisha 61922, P. O. Box 199 Saudi Arabia; University of Manouba, ISBST, BVBGR-LR11ES31, Biotechpole Sidi Thabet 2020 Ariana Tunisia

## Abstract

The present study evaluates the chemical profiling of the essential oil of a halophyte, *L. maritima* (*Lm*EO), and its protective potential against CCl_4_-induced oxidative stress in rats. Forty compounds have been identified in *Lm*EO. The major components are α-pinene (3.51%), benzyl alcohol (8.65%), linalool (22.43%), pulegone (3.33%), 1-phenyl butanone (7.33%), globulol (4.32%), γ-terpinene (6.15%), terpinen-4-ol (4.31%), α-terpineol (3.9%), ledol (3.59%), *epi*-α-cadinol (3.05%) and α-cadinol (4.91%). In comparison with the CCl_4_-intoxicated group, *Lm*EO treatment resulted in decreased liver serum marker enzymes, decreased lipid peroxidation and increased antioxidant enzyme levels, with overall further amelioration of oxidative stress. The administration of *Lm*EO to CCl_4_-treated rats at a dose of 250 mg kg^−1^ body weight significantly reduced the toxic effects and the oxidative stress on the liver, thus validating the traditional medicinal claim of this plant. Moreover, the anti-inflammatory activity of *Lm*EO was evaluated in lipopolysaccharide-stimulated murine RAW 264.7 cells. Our oil could modulate the inflammatory mode of the macrophages by causing reduction in iNOS and COX_2_ enzymes as well as in IL-1β, IL-6, and TNF-α cytokine levels. These findings suggest that *Lm*EO exerts anti-inflammatory effects by regulating the expression of inflammatory cytokines.

## Abstract 2

The present study evaluates the chemical profiling of the essential oil of a halophyte, *L. maritima* (*Lm*EO), and its protective potential against CCl_4_-induced oxidative stress in rats.

## Graphical abstract

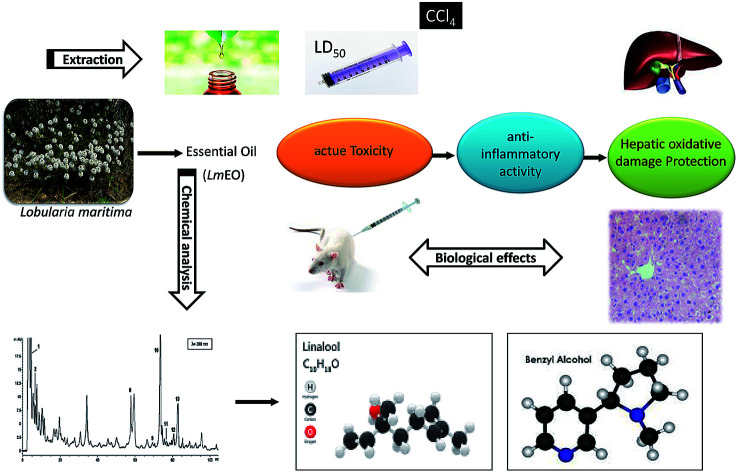

## Introduction

The liver is a very important organ which is responsible for metabolic and secretor activities. It is a sensitive target site for substances that modulate biotransformation. Furthermore, it plays a key role in body detoxification from exogenous and endogenous challenges, such as xenobiotics, drugs, viral infections and chronic alcoholism.^1^ The carbon tetrachloride (CCl_4_)-induced hepatotoxicity model is extensively used to evaluate the antioxidant effects of drugs and plant extracts.^2^ CCl_4_ accumulates in hepatic parenchyma cells and is metabolized into 
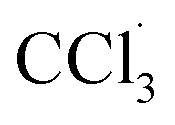
 radicals by liver cytochrome P450-dependent monooxygenases.^3^ It causes oxidative stress and accumulation of reactive oxygen species (ROS). ROS, including superoxide anions (O_2_˙^−^), hydrogen peroxide (H_2_O_2_), hydroxyl radicals 
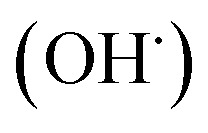
 and peroxyl radical 
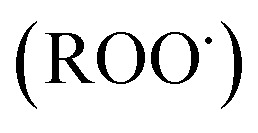
, have been recognized to stimulate tissue oxidative damage and cause several diseases, such as atherosclerosis, diabetes mellitus, cancer, and neurodegenerative diseases, as well as ageing processes.^4^ In addition to these conditions, oxidative stress is involved in liver pathologies and mostly results in progressive evolution of fatty liver, necrosis, fibrosis, cirrhosis and hepatocellular carcinoma.

Oxidative stress can result from excess ROS production and/or deficient antioxidant capacity. Several chemical drugs have been implicated in the aetiology of liver oxidative-stress diseases. In fact, CCl_4_ is a toxic substance used to induce liver damage in rats.^5^ CCl_4_ catabolic radicals induce lipid peroxidation and damage liver cell membranes and organelles, causing swelling and necrosis. Therefore, CCl_4_, a hepatotoxin used to evaluate the hepatoprotective potential of plant extracts, is commonly used to induce liver damage by producing free radical intermediates (malondialdehyde and 4-hydroxy-2-nonenal). These radicals quickly add molecular oxygen to form highly reactive trichloromethyl peroxy radicals, CCl_3_OO˙, which react with proteins and lipids.^3^ They remove hydrogen atoms from unsaturated lipids; this initiates lipid peroxidation, which has the ability to cause structural and functional injury to cell membranes and thus increase membrane permeability, leading to leakage of hepatic enzymes (ALT, AST, ALP, and LDH).^6^ Biochemically, hepatocellular damage occurs due to high levels of cytosolic enzymes, such as AST, ALT and ALP, in the blood circulation.^7^

In addition to oxidative stress, inflammation is a common biological mechanism contributing to compromised liver function. Inflammation is a physiological body response to stimuli, including infections and tissue injury. Macrophages, which are ubiquitously distributed in tissues *via* the mononuclear phagocyte system, play an essential role in innate immunity and are also involved in both host defense mechanism and inflammation.^8^ They support homeostasis and host defense against intracellular parasitic bacteria and pathogenic protozoa.^9^ When activated, macrophages are responsible for excessive secretion of proinflammatory enzymes, such as inducible NO synthase (iNOS) and cyclooxygenase (COX-2).^10^ However, overproduction of these mediators can cause harmful effects to tissues. These effects have been reported to be associated with the pathogenesis of various inflammatory-related diseases, such as rheumatoid arthritis, diabetes, inflammatory bowel disease, atherosclerosis, and cancer.^11^

Lipopolysaccharide (LPS), a component of the outer membrane of Gram-negative bacteria, stimulates macrophage activation.^12^ Activated macrophages play an important role in inflammatory response by producing cytokines, including interleukin-1 beta (IL-1β), interleukin-6 (IL-6), and tumor necrosis factor-alpha (TNF-α).^9^ Inflammatory response is a crucial protective attempt of the host defense to remove injurious stimuli and initiate healing. Despite this protective role, the overproduction of inflammatory mediators can be a major cause of tissue damage, sepsis, and cancer.^13^

ROS overproduction and cell redox imbalance play key roles in the pathophysiology of the inflammatory response.^14^ Substantial evidence implicates oxidative stress as an important pathogenic factor in inflammatory response.^15^ ROS are intracellularly generated from several sources, including mitochondrial respiration, cytochrome P450, and the nicotinamide adenine dinucleotide phosphate (NADPH) oxidase system.^16^ Phagocyte NADPH oxidase 2 (NOX2) is the major source of ROS generation in the macrophage response to LPS.^17^ Therefore, LPS may induce an inflammatory response through NADPH oxidase activation-driven oxidative stress. Likewise, the inhibition of ROS production using inhibitors of NADPH oxidase has been proposed as an alternative approach to conventional antioxidant therapies against LPS-induced inflammation. In ROS-induced liver diseases, exogenous antioxidative compounds must be delivered to maintain balance between oxidants and antioxidants in order to prevent subsequent pathologies. However, conventional and synthetic drugs used in treating liver diseases are inadequate to some extent and may cause serious adverse effects.^18^ For this reason, a considerable number of liver patients prefer safer approaches, such as using more effective natural antioxidants. Accordingly, plant extracts and their derived metabolites, such as phenolic compounds, offer an opportunity in this respect.^19^ The use of natural antioxidants has been proposed as a therapeutic regime and as drug co-adjutants in liver damage treatments.

Plant secondary metabolites are reputed for their anti-inflammatory properties.^20,21^ Among these metabolites, essential oils (EOs) are well-known for their antioxidant properties. These oils have been widely used in traditional medicine to treat inflammatory diseases.^22,23^ They are also exploited as natural additives or ingredients in foods which claim beneficial health properties.^24^

*Lobularia maritima* (*Alyssum martima*, Brassicaceae), commonly known as sweet alyssum, is an annual ornamental halophyte that is commonly found along the Tunisian seashore. It can tolerate salinity up to 400 mM NaCl.^25^

To our knowledge, the chemical composition and biological activities of the halophyte *L. maritima* have not been reported. Moreover, the use of this plant to alleviate the oxidative damage induced by CCl_4_ has not been previously explored. This work is the first investigation dealing with the (i) chemical identification of the bioactive compounds extracted from *L. maritima* using gas chromatography-mass spectrometry (GC-MS); (ii) the investigation of the *in vitro* antioxidant effects of *Lm*EO in several *in vitro* systems; (iii) the anti-inflammatory mechanism of *Lm*EO in LPS-induced murine macrophage cells; and (iv) the exploration of the possible protective effects of *Lm*EO against liver oxidative damage following intraperitoneal administration of CCl_4_ by assessing the oxidative stress profile and some serum biochemical parameters.

## Materials and methods

### Plant material

The aerial parts of the halophyte *Lobularia maritima (L. maritima)* (flowering stage) were collected in April 2017 in Chebba, Mahdia in Tunisia. The aerial parts were air-dried at room temperature in shade. After that, they were finely ground and maintained in a sealed bag in a cold, dry place until they were used.

### Essential oil isolation

The oil extraction was performed on 1 kg of fresh *L. maritima* leaves by steam distillation for 3 h using a Clevenger-type apparatus. The aqueous phase was extracted with dichloromethane (3 × 50 mL) and dried with anhydrous sodium sulphate. The resulting *Lm*EO was stored at 4 °C prior to further analysis.^26^*Lm*EO was solubilized in *n*-hexane for GC-MS analysis. The oil extraction yield was calculated according to the following formula: oil (% v/w) = volume of *Lm*EO (mL)/weight of raw material (g).

### Gas chromatography-mass spectrometry (GC-MS)

GC/MS analysis was carried out using a Shimadzu QP2010SE 15A operating at 70 eV ionization energy, equipped with a Rtx-5MS column (phenyl methyl siloxane 30 m × 0.25 mm, 0.25 μm film thickness) with He as the carrier gas at a flow rate of 0.9 mL min^−1^ and a split ratio of 1 : 20. The acquisition mass range was 35 to 300 and the scan time was 0.5 s/scan. Retention indices were specified using retention times of *n*-alkanes that were injected after the oil under the same chromatographic conditions. The retention indices for all components were specified according to the Van Den Dool method using *n*-alkanes as a standard. The compounds were identified by comparison of their retention indices (RI, Rtx-5MS) with those reported in the literature and by comparison of their mass spectra with the Wiley and NIST libraries or with published mass spectra.^26^

### Antioxidant testing assays

#### DPPH radical scavenging activity

The radical scavenging activities of the different fractions were determined using 1,1-diphenyl-2-picrylhydrazyl (DPPH) radical as a reagent according to the method of Kirby and Schmidt with some modifications.^27^ Briefly, 1 mL of a 4% (w/v) solution of DPPH radical in ethanol was mixed with 500 μL of sample solution (different concentrations). The mixture was incubated for 20 min in the dark at room temperature. The scavenging capacity was read spectrophotometrically by monitoring the decrease of the absorbance at 517 nm. Lower absorbance of the reaction mixture indicates higher free radical scavenging activity. Ascorbic acid was used as a standard. The percent DPPH scavenging effect was calculated using the following equation: DPPH scavenging effect (%) = (*A*_control_ − *A*_sample_/*A*_control_) × 100; *A*_control_ is the absorbance of the control reaction, where the sample is replaced by 500 μL ethanol. Tests were carried out in triplicate.

#### β-Carotene bleaching assay

The antioxidant activity was determined according to the β-carotene bleaching method described by Pratt.^28^ A stock solution of a β-carotene/linoleic acid mixture was prepared as follows: 0.5 mg of β-carotene was dissolved in 1 mL of chloroform with 25 μL of linoleic acid and 200 mg of Tween-20. The chloroform was completely evaporated using a vacuum evaporator. Then, 100 mL of distilled water saturated with oxygen (30 min) were added, and the obtained solution was vigorously shaken. 4 mL of this reaction mixture were dispensed into test tubes, and 200 μL of each sample, prepared at different concentrations, were added. The emulsion system was incubated for 2 h at 50 °C. The same procedure was repeated with butylhydroxytoluene (BHT) as a positive control and a blank without sample as a negative control. After this incubation period, the absorbance of each mixture was measured at 490 nm. The antioxidant activity in the β-carotene bleaching model as a percentage (*A*%) was calculated with the following equation: 
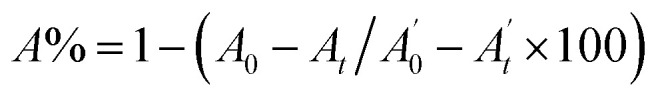
, where *A*_0_ and 
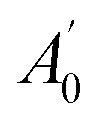
 are the absorbances of the sample and the blank, respectively, measured at zero time, and *A*_*t*_ and 
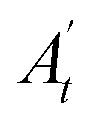
 are the absorbances of the sample and the blank, respectively, measured after 2 h. All tests were carried out in triplicate.

### *In vivo* antioxidant properties

#### Animals

Wistar rats weighing 200 to 220 g were obtained from the Central Pharmacy of Tunis. They were kept in cages in a breeding farm at a temperature of 21 ± 1 °C with alternating periods of 14 h darkness and 10 h illumination and a relative humidity of around 40%. All rats had free access to drinking water and food. The pelleted diet for rats was 15% protein and was supplied by the Industrial Society of Concentrate (SICO, Sfax, Tunisia). The experimental protocol was approved by the Ethical Committee of the Faculty of Sciences of Sfax with ethics approval number 1204. All the experimental procedures were carried out in accordance with international guidelines for the care and use of living animals in scientific investigations.^29^

#### Acute toxicity studies: lethal dose 50 (LD_50_)

Acute toxicity studies were performed for *Lm*EO in male Wistar rats as per OECD guidelines. A single dose of the oil was administered orally to each animal. The animals were fasted overnight and provided only water, after which the rats were treated with graded doses of *Lm*EO (100 mg kg^−1^, 200 mg kg^−1^, 400 mg kg^−1^ and 5000 mg kg^−1^, i.g.) and observed for 14 days to assess the acute oral toxicity of *Lm*EO. The animals were observed individually during the first 30 min and thereafter every 24 hours for a period of 14 days.^6,26^

### Experimental design

Rats were randomly assigned to four groups of eight animals each. Animals in the first group receiving distilled water and a standard laboratory diet served as controls (C). The second group (CCl_4_), the hepatoxicity model, was given a single dose of CCl_4_ (1 mL kg^−1^ in 1% olive oil i.p.) on the 14^th^ day.^6,26^ These doses were selected on the basis of previous studies which did not reveal any toxic effects in adult rats and were effective against toxicity. Animals in the third group (*Lm*EO) were given a daily i.p. injection of *Lm*EO at 250 mL kg^−1^ b.w. and distilled water as sole beverage for 15 days. The fourth group (*Lm*EO + CCl_4_) was pretreated with *Lm*EO and intoxicated with CCl_4_ on the 14^th^ day.^6,26^ The CCl_4_ dose was selected according to the chronic oral reference dose recommended for CCl_4_ (CASRN 56-23-5).^30^ During the 2 weeks of the experimental period, all animals survived.

### Organ sampling

At the end of the experimental period (15 days), 24 h after the administration of CCl_4_, control and treated rats were anesthetized with chloral hydrate by intra-abdominal injection. The body weights of the rats were recorded, and blood samples from the brachial artery were collected in heparin tubes. At the end of the experimental period, the animals in the different groups were killed by cervical decapitation to avoid animal stress. Plasma samples were obtained from blood after centrifugation at 2500 × *g* for 15 min to estimate selected serum biochemical parameters. The samples were maintained at −20 °C until analysis. All samples were analyzed in triplicate.

The livers of the rats were collected, cleaned and weighed. Some samples were homogenized (1 : 2, w/v) in 50 mmol L^−1^ Tris buffer (pH 7.4) containing 150 mmol L^−1^ of NaCl using an ultra-Turrax device. The homogenates were centrifuged at 5000 × *g* for 25 min at 4 °C, and aliquots of the supernatant were maintained at −20 °C until the analyses.

In parallel, portions of the livers were immediately fixed in Bouin solution (saturated picric acid added to 37% to 40% formaldehyde and glacial acetic acid, 75 : 25 : 5 v/v) for histological studies.^6,26^

### Serum parameters

Serum samples were obtained by centrifugation of blood at 2700 × *g* for 15 min at 4 °C and were then divided into Eppendorf tubes. Isolated sera were stored at −20 °C until they were used for further analyses. The levels of serum ALT, AST, ALP, and lactate dehydrogenase (LDH) were measured using commercial kits according to the manufacturer's directions (Biolabo, Maizy, France) on an automatic biochemistry analyzer (Vitalab Flexor E, Diamond Diagnostics, Holliston, MA).

### Biochemical assays

#### Protein quantification

Protein content was evaluated as described by Lowry *et al.* using bovine serum albumin (BSA) as a standard.^31^

#### Measurement of lipid peroxidation

The formation of lipid peroxides was measured in the liver. The formation of malondialdehyde (MDA), a product of fatty acid (FA) peroxidation, was measured spectrophotometrically at 532 nm using a thiobarbituric acid reactive substance (TBARS), essentially using the method described by Yagi.^32^ Briefly, an aliquot of liver and kidney extracts supernatant was mixed with 1 mL of 5% trichloroacetic acid and centrifuged at 2500 × *g* for 10 min. One mL of thiobarbituric acid reagent (0.67%) was added to 500 μL of supernatant, and the sample was heated at 90 °C for 15 min. The mixture was cooled and the absorbance was measured at 532 nm using a spectrophotometer (Jenway UV-6305, Essex, England). The malondialdehyde values were calculated using 1,1,3,3-tetraethoxypropane as a standard and were expressed as nmol of malondialdehyde per mg of protein.

#### Antioxidant enzyme studies

In liver tissues, the SOD activity was determined according to the colorimetric method of Beyer and Fridovich^33^ using the oxidizing reaction of nitroblue tetrazolium (NBT); CAT activity was measured by the UV colorimetric method of Aebi^34^ using H_2_O_2_ as the substrate; glutathione peroxidase (GPx) activity was measured by a modification of the colorimetric method of Floke and Gunzler using H_2_O_2_ as the substrate and reduced GSH.^35^

#### Histopathological examination

After fixation in Bouin solution, pieces of fixed tissue were embedded into paraffin, cut into 5 μm slices and colored with hematoxylin–eosin to examine the tissue constitution.^33^ Six slices were prepared from each liver. All sections were evaluated semi-quantitatively to determine the degree of liver injury. The steatohepatitis calculation system was applied to evaluate necrosis, inflammation, and ballooning.^36^

#### Cell culture

RAW 264.7 cells belonging to a murine macrophage cell line were purchased from the American Type Culture Collection (Teddington, UK). These cells (2 × 10^5^) were cultured in a 96-well plate containing Dulbecco's modified Eagle's medium (Sigma) and supplemented with 10% fetal bovine serum in a CO_2_ incubator (5% CO_2_) at 37 °C.

#### Cell viability

Cells were preincubated for 24 h in a CO_2_ incubator, pretreated with three *Lm*EO concentrations (0 μg mL^−1^, 20 μg mL^−1^, 40 μg mL^−1^ and 80 μg mL^−1^) for 1 h, and co-stimulated with 0.5 μg mL^−1^ lipopolysaccharide (LPS) for 24 h at 37 °C. Afterwards, the cells were washed twice with phosphate-buffered saline (PBS). 10 μL of 5 mg mL^−1^ 3-(4,5-dimethylthiazol-2-yl)-2,5-diphenyltetrazolium bromide (MTT; Sigma-Aldrich) was added, followed by 100 μL of a mixture of 0.04 N HCl in isopropanol/Triton X-100 to dissolve blue formazan crystals, 2 h later.^6^ The absorbance was measured using a spectrophotometer (Biomate 5, Thermo Electron Corporation, Waltham, MA, USA) at the wavelength of 490 nm. The effects of *Lm*EO and LPS on the cell viability were assessed as the percentage of viable cells compared with the vehicle-treated control cells (which received cell-grade DMSO at a safe concentration), which were arbitrarily assigned a viability of 100%.

#### Measurement of nitric oxide, TNF-α, IL-1β, IL-6, and IL-10 levels

RAW 264.7 cells were placed in a 12-well plate at a density of 2 × 10^5^ cells per well and incubated for 24 h. The cultured cells were treated with various *Lm*EO concentrations for 1 h and stimulated with 0.5 mg mL^−1^ LPS for 24 h. The cultured media were collected after centrifugation at 2000 × *g* for 10 min and stored at −80 °C until analysis.

The nitrite concentration in the cultured media was measured as an indicator of nitric oxide (NO) production based on the Griess reaction.^37^ The nitrite concentration was calculated using a sodium nitrite standard curve. The percentage of NO inhibition in the treated cells was compared to LPS-only treated cells (100%), and the half NO inhibitory concentrations (IC_50_) for various *Lm*EO concentrations were determined.

The levels of IL-1β, IL-6, IL-10, and TNF-α in the culture media were quantitated by ELISA (R&D Systems) in accordance with the manufacturer's instructions.

#### Western blot analysis

RAW 264.7 cells placed in a 12-well plate were pretreated with various *Lm*EO concentrations for 1 h and stimulated with LPS for 6 h. After the incubation period, the cells were scraped from the flasks and lysed in lysis buffer. The samples were boiled at 100 °C for 5 min and centrifuged for 2 min at 4 °C. Protein extracts were run on 10% sodium dodecyl sulfate polyacrylamide gel electrophoresis gels and transferred onto polyvinylidene difluoride membranes (Millipore, Boston, MA, USA). The membranes were blocked with 5% nonfat dry milk in Tris-buffered saline and Tween 20 buffer for 1 h at room temperature. Afterward, the membranes were incubated with an appropriate dilution ratio of the relative primary antibody overnight at 4 °C. The membranes were further incubated with the secondary antibody for 4 h at room temperature and detected using an enhanced chemiluminescence reagent. The membranes were washed three times and the immunoreactive proteins were detected with an enhanced chemiluminescence system (GE Healthcare, Little Chalfont, Buckinghamshire, UK).

The results of the western blot analysis were quantified by measuring the relative intensity compared to the control and are represented by relative intensities. Mouse β-actin was simultaneously detected as an internal control to monitor the intensity. The bands for the iNOS, COX-2, and β-actin antibodies were recognized at ∼135 kDA, ∼72 kDA, and ∼45 kDa, respectively.

#### Statistical analysis

All values are expressed as mean ± SEM for continuous variables or as the median with the interquartile range [25%, 75%] where appropriate. The results were analyzed by one-way analysis of variance (ANOVA) followed by Tukey test for multiple comparisons using SPSS for Windows (version 12) or ANOVA-on-ranks with Dunn's correction. Differences were considered significant at *P* < 0.05.

## Results

### *Lm*EO chemical constitution

The hydrodistillation of *L. maritima* aerial parts generated a yellow oil (yield of 2.4%, v/w). Upon GC/MS analysis, *Lm*EO was found to contain 40 constituents, with 90.55% identified constituents (Table 1). The volatile oil contained 74.40% oxygenated monoterpenes and 16.15% monoterpene hydrocarbons. The major components were α-pinene (3.51%), benzyl alcohol (8.65%), linalool (22.43%), α-terpineol (3.9%), pulegone (3.33%), 1-phenyl butanone (7.33%), ledol (3.59%), globulol (4.32%) and α-cadinol (4.91%). It is important to note that the oxygenated monoterpene fraction was present in a relatively high amount (>74.40%).

**Table 1:** Chemical constituents of *Lobularia maritima* essential oil (*Lm*EO) with the percentages of the contents obtained by hydrodistillation

No.	Compound^a^	KI^b^	%^c^
1	Furfural	800	0.18
2	α-Thujene	883	0.12
3	α-Pinene	938	3.51
4	Sabinene	976	2.13
5	Myrcene	947	0.15
8	α-Phellandrene	1003	0.37
9	δ-3-Carene	1016	0.15
10	Benzyl alcohol	1040	8.65
11	γ-Terpinene	1052	6.15
12	Acetophenone	1065	0.15
13	*Z*-Linalool oxide (furanoid)	1074	0.16
14	Linalool	1082	22.43
15	1-Terpineol	114 3	5.6
16	Terpinen-4-ol	1175	4.31
17	α-Terpineol	1176	3.9
18	Pulegone	1238	3.33
19	δ-Elemene	1338	0.22
20	Isoledene	1376	0.37
21	α-Copaene	1379	0.22
22	β-Bournonen	1380	0.26
23	β-Cubebene	1384	0.24
24	α-Gurjunene	1406	0.22
25	1-Phenyl butanone	1425	7.33
26	α-Humulene	1455	0.15
27	Germacrene D	1462	0.42
28	β-Selinene	1484	0.24
29	β-Sesquiphellandrene	1501	0.17
30	Germacrene B	1535	0.34
31	Ledol	1561	3.59
32	Germacrene D-4-ol	1573	0.12
33	Spathulenol	1576	0.47
34	Globulol	1590	4.32
35	1.10-di-*epi*-Cubenol	1627	1.67
36	10-*epi*-γ-Eudesmol	1635	0.27
37	*epi*-α-Cadinol	1643	3.05
38	α-Cadinol	1672	4.91
39	*Z*-Methyl epijasmonate	1675	0.14
40	*Z*-α-Bisabolene epoxide	1680	0.54
Monoterpene hydrocarbons	—	—	16.15
Oxygenated monoterpenes	—	—	74.40
Total (%)			90.55

a Identification of components based on GC-MS Wiley version 7.0 library data and National Institute of Standards and Technology 05 MS (NIST) library data.

b KI: Kovats indices on an HP-5MS capillary column in reference to C_10_–C_22_*n*-alkanes injected under the same conditions.

c %: percentages are the means of two runs and were obtained from electronic integration measurements using a selective mass detector.

### *In vitro* antioxidant properties

#### Antioxidant capacities of *Lm*EO

**Fig. 1:**
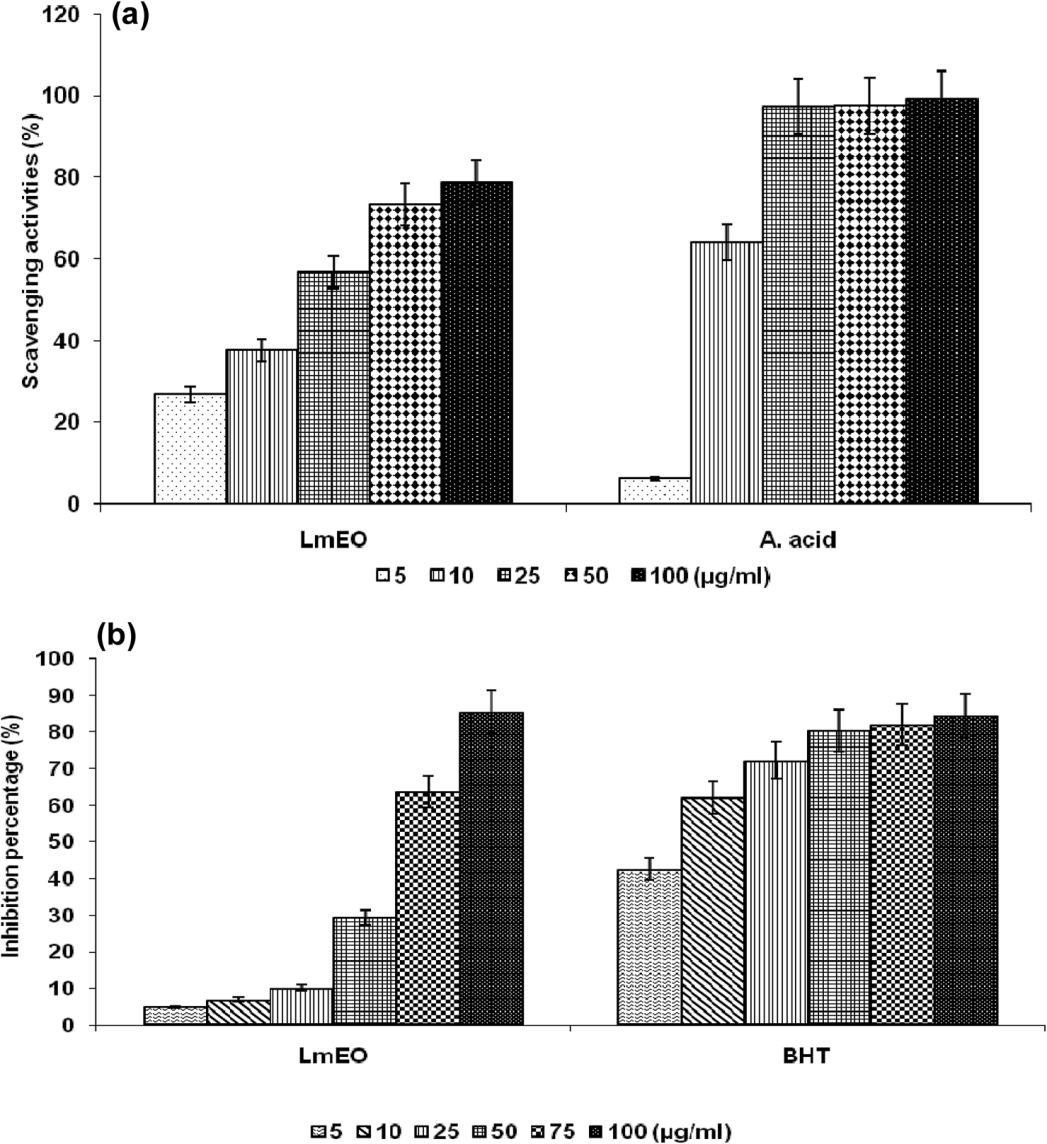
(a) Scavenging effects of *Lm*EO at different concentrations of 5, 10, 25, 50 and 100 μg mL^−1^ on the stable 1,1-diphenyl-2-picrylhydrazyl radical (DPPH). Results are expressed as percentage of decrement of absorbance at 517 nm with respect to the control. Ascorbic acid was used as a standard. (b) Antioxidant activities of *Lm*EO at different concentrations of 5, 10, 25, 50, 75 and 50 μg mL^−1^ measured by the β-carotene bleaching method. Butylhydroxytoluene (BHT) was used as a standard. The results presented are representative of three independent experiments. Values are expressed as mean ± SEM (*n* = 3).

The DPPH test aims to measure the capacity of plant extracts and molecules to scavenge the stable free radical DPPH by donation of a hydrogen atom or an electron. If the extract has the capacity to scavenge the DPPH free radical, the initial blue/purple solution will become yellow due to the formation of diphenylpicrylhydrazine. The effect of the *Lm*EO on DPPH radical scavenging was compared to that of ascorbic acid, used as a positive control, and appreciated by the determination of the IC_50_ values. As shown in Fig. 1a, the DPPH tests revealed that an increase in the tested concentration resulted in an increase in free radical-scavenging activity in a dose-dependent manner. The current study demonstrates that *Lm*EO exhibited strong radical scavenging activity compared to the standard ascorbic acid.

For the β-carotene bleaching method, the inhibitory effects of *Lm*EO on lipid peroxidation were determined by the β-carotene/linoleic acid bleaching test. Fig. 1b shows various degrees of linoleic acid oxidation and subsequent β-carotene bleaching after addition of *Lm*EO and BHT used as a positive control at different concentrations. This antioxidant activity was dose dependent, as found in the DPPH tests.

#### Acute toxicity studies

*Lm*EO did not show any signs or symptoms of toxicity and mortality up to 2000 mg kg^−1^ dose.

#### Serum biochemical enzyme markers

**Fig. 2:**
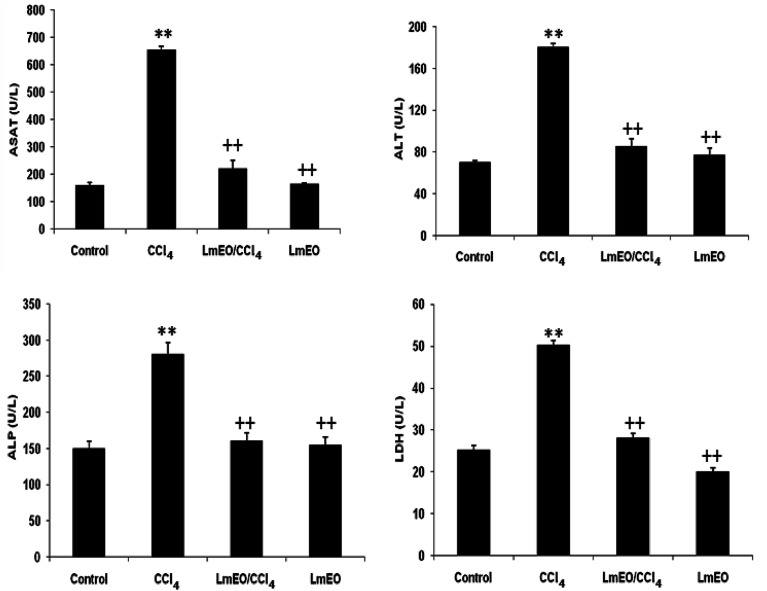
Plasma levels of bio-indices of liver functions in adult rats treated with CCl_4_ alone or concomitantly with *Lm*EO for 15 days; C: control; CCl_4_: carbon tetrachloride; (CCl_4_ + *Lm*EO): rats pre-treated with *Lm*EO and intoxicated with CCl_4_ at 14 days; aspartate aminotransferase (AST); alanine aminotransferase (ALT); alkaline phosphatase (ALP); lactate dehydrogenase (LDH). Values are expressed as mean ± SEM of eight animals in each group. One-way ANOVA followed by Tukey test for comparison between groups: comparison between CCl_4_ and control groups: **P < 0.01; comparison between CCl_4_ + *Lm*EO and CCl_4_ groups: ^++^*P* < 0.01.

The activities of various biochemical enzymes in the control, CCl_4_-intoxicated and *Lm*EO-treated animal groups are presented in Fig. 2. The serum levels of AST, ALT, ALP and LDH were significantly higher in the CCl_4_-treated animals (group II) compared to the control rats. Pre-treatment with *Lm*EO at a dose of 250 mg kg^−1^ BW daily for 15 days maintained the levels of these enzymes around normal values in the experimental group IV rats intoxicated with CCl_4_.

#### Effects on lipid peroxidation

**Fig. 3:**
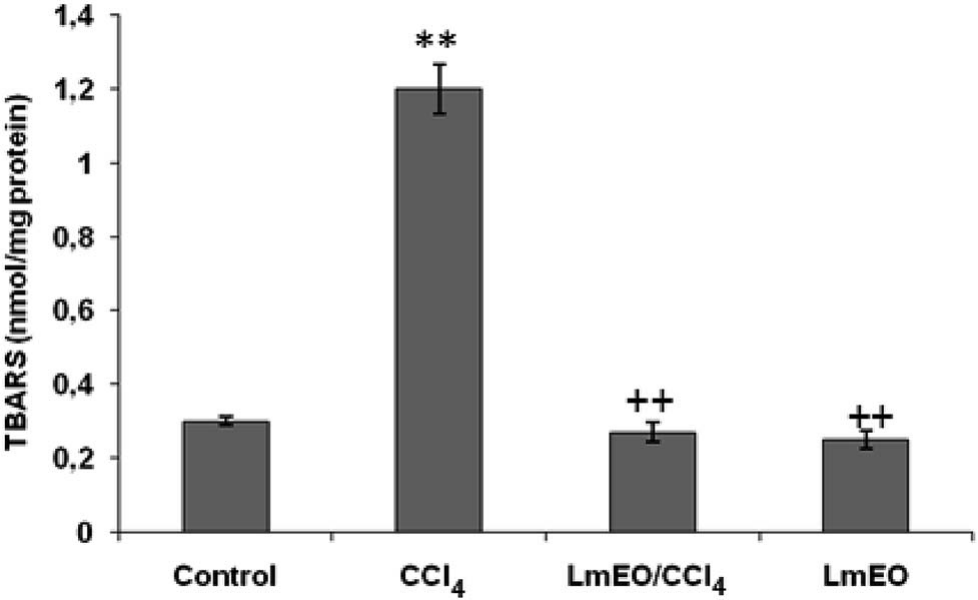
Effects of CCl_4_, *Lm*EO and their combinations (*Lm*EO/CCl_4_) on hepatic TBARS of control and experimental rats. Values are expressed as mean ± SEM of eight animals in each group. One-way ANOVA followed by Tukey test for comparison between groups: comparison between CCl_4_ and control groups: **P < 0.01; comparison between CCl_4_ + *Lm*EO and CCl_4_ groups: ^++^*P* < 0.01.

The effects of *Lm*EO on CCl_4_-induced lipid peroxidation in the liver are shown in Fig. 3. CCl_4_ increased the hepatic TBARS concentration significantly. Hepatic TBARS was inhibited by *Lm*EO pretreatment. Single dose pretreatment (250 mg kg^−1^ b.w.) was the most efficient in inhibiting hepatic lipid peroxidation. No significant differences in the TBARS level were observed in rats treated with *Lm*EO only (group III) compared with the control value.

#### Effects on antioxidant enzymes

**Fig. 4:**
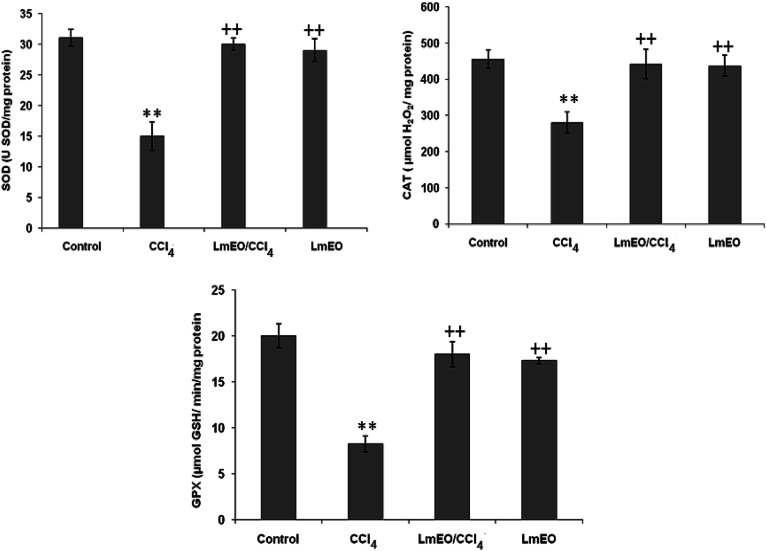
Effects of CCl_4_, *Lm*EO and their combination *Lm*EO/CCl_4_ on the activities of enzymatic antioxidants in the livers of control and experimental rats. C: control; CCl_4_: carbon tetrachloride; (CCl_4_ + *Lm*EO): rats pre-treated with *Lm*EO and intoxicated with CCl_4_ at 14 days; SOD: superoxide dismutase (U SOD per mg protein); CAT: catalase (mmol per mg protein); GPx: glutathione peroxidase (nmol per mg protein). Values are expressed as mean ± SEM of eight animals in each group. One-way ANOVA followed by Tukey test for comparison between groups: comparison between CCl_4_ and control groups: **P < 0.01; comparison between CCl_4_ + *Lm*EO and CCl_4_ groups: ^++^*P* < 0.01.

SOD, CAT and GPx were measured as indices for the antioxidant status of liver tissues (Fig. 4). These enzyme activities were significantly lower (*p* < 0.05) in the livers of CCL_4_-treated group II rats (19 ± 0.4 units per mg protein, 290 ± 10 μmol H_2_O_2_ per mg protein, 12 ± 0.6 μmol GSH per min per mg protein, respectively) compared with their corresponding controls (31 ± 1 units per mg protein, 445 ± 15 μmol H_2_O_2_ per mg protein, 19.35 ± 0.24 μmol GSH per min per mg protein, respectively). *Lm*EO (250 mg kg^−1^ b.w.) alone did not affect the levels of any of the tested oxidative enzymes. However, in its combined form with CCl_4_, it resulted in restoration of all antioxidant enzymes to their control levels (30 ± 1 units per mg protein, 442 ± 12 μmol H_2_O_2_ per mg protein, and 18 ± 0.4 μmol GSH per min per mg protein for SOD, CAT and GPx, respectively) in group IV rats.

### Histopathological findings

**Fig. 5:**
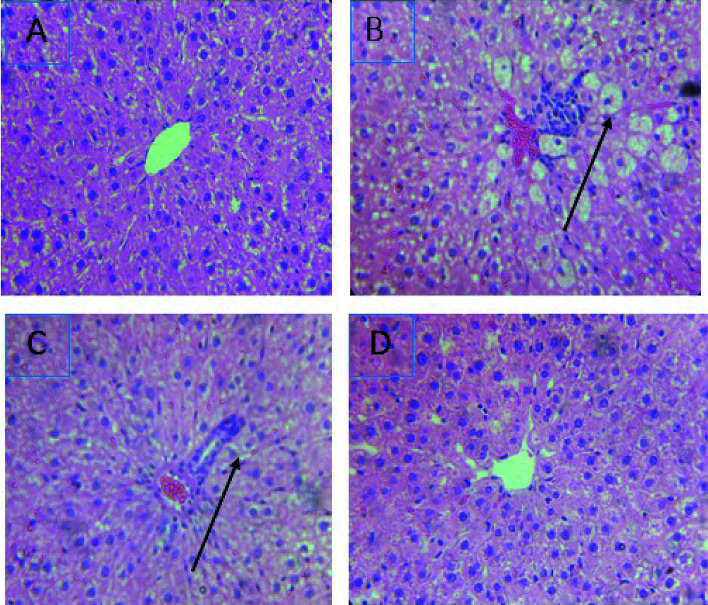
Effects of *Lm*EO on the histological morphology of rat liver fibrosis with Masson staining (×100). (A) Control group; (B) *Lm*EO group; (C) CCl_4_-treated group; (D) CCl_4_ + *Lm*EO group.

The treatment with CCl_4_ caused excessive necrosis associated with neutrophilic infiltration, which is frequently observed in the case of swelling of liver cells, as well as neutrophilic infiltration (arrows) and several ballooning degenerations of hepatocytes (Fig. 5). However, according to the microscopic examinations, the severe hepatic lesions induced by CCl_4_ were considerably reduced by the administration of *Lm*EO (250 mg kg^−1^ BW) (Fig. 5). These data are well correlated with those of the serum biochemical parameters and oxidative stress markers. Necrosis was markedly reduced and minimized by pre-treatment with 250 mg kg^−1^ of *Lm*EO.

#### Effects of *L. maritima* essential oil on cell viability

**Fig. 6:**
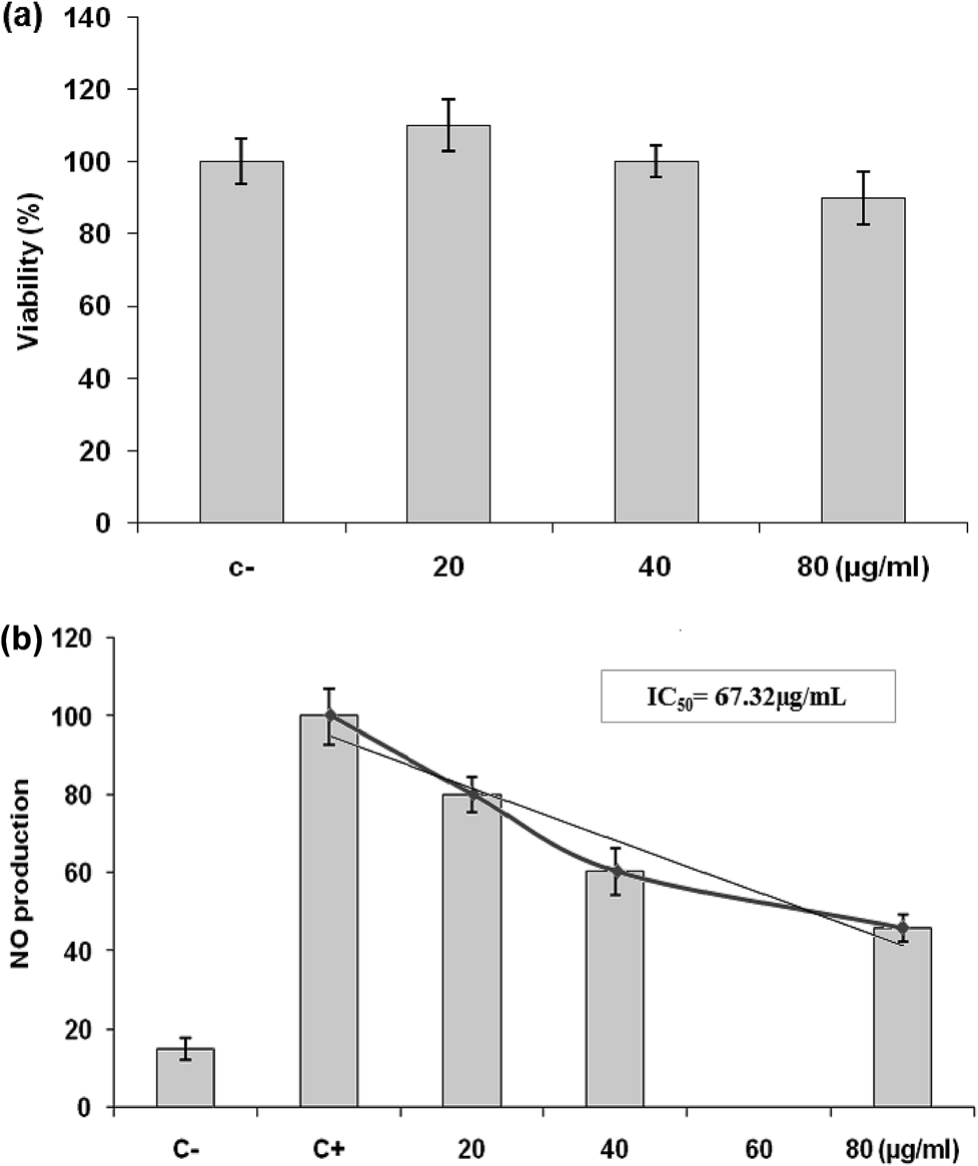
(a) Effects of *Lm*EO on the viability of RAW 264.7 cells determined by MTT assay. The cells were treated with different concentrations of *Lm*EO for 24 h. (C−): negative control values were obtained in the absence of components. None of the tested concentrations of *Lm*EO showed significant detrimental effects. (b) Effects of *Lm*EO on nitric oxide production (NO) in lipopolysaccharide-stimulated macrophages. Cells were treated with lipopolysaccharide and different concentrations of *Lm*EO for 24 h. The supernatants of the cultured cells were examined for NO levels by the Griess method. (C−): negative control values were obtained in the absence of lipopolysaccharide and the essential oil. (C+): the positive control was lipopolysaccharide-only treated cells. Data represent mean ± SEM of three independent experiments.*P < 0.05 compared with the LPS-only treatment group, **P < 0.01 *vs.* the positive control. Ctrl = control.

We used the MTT colorimetric assay to study the effects of different concentrations of *Lm*EO on the growth of RAW 264.7 cells. Fig. 6a shows that *Lm*EO had no significant cytotoxic effects on the cells at concentrations of 20 to 80 μg mL^−1^. Therefore, we used the oil at concentrations of 20 to 80 μg mL^−1^ in subsequent experiments.

#### Effects of *L. maritima* essential oil on the production of NO in LPS-stimulated RAW 264.7 cells

We investigated the potential anti-inflammatory effects of *Lm*EO by evaluating the production of NO in LPS-stimulated RAW 264.7 cells. As shown in Fig. 6b, NO production was substantially higher in the LPS-treated cells than in the untreated cells. However, *Lm*EO suppressed NO production in the LPS-treated cells in a dose-dependent manner, with a concentration required for 50% inhibition (IC_50_) of 67.32 μg mL^−1^. Addition of 80 μg mL^−1^*Lm*EO to the cells caused a reduction in LPS-induced NO production by 45%.

#### Effects of *Lm*EO on LPS-induced inflammatory cytokines

**Fig. 7:**
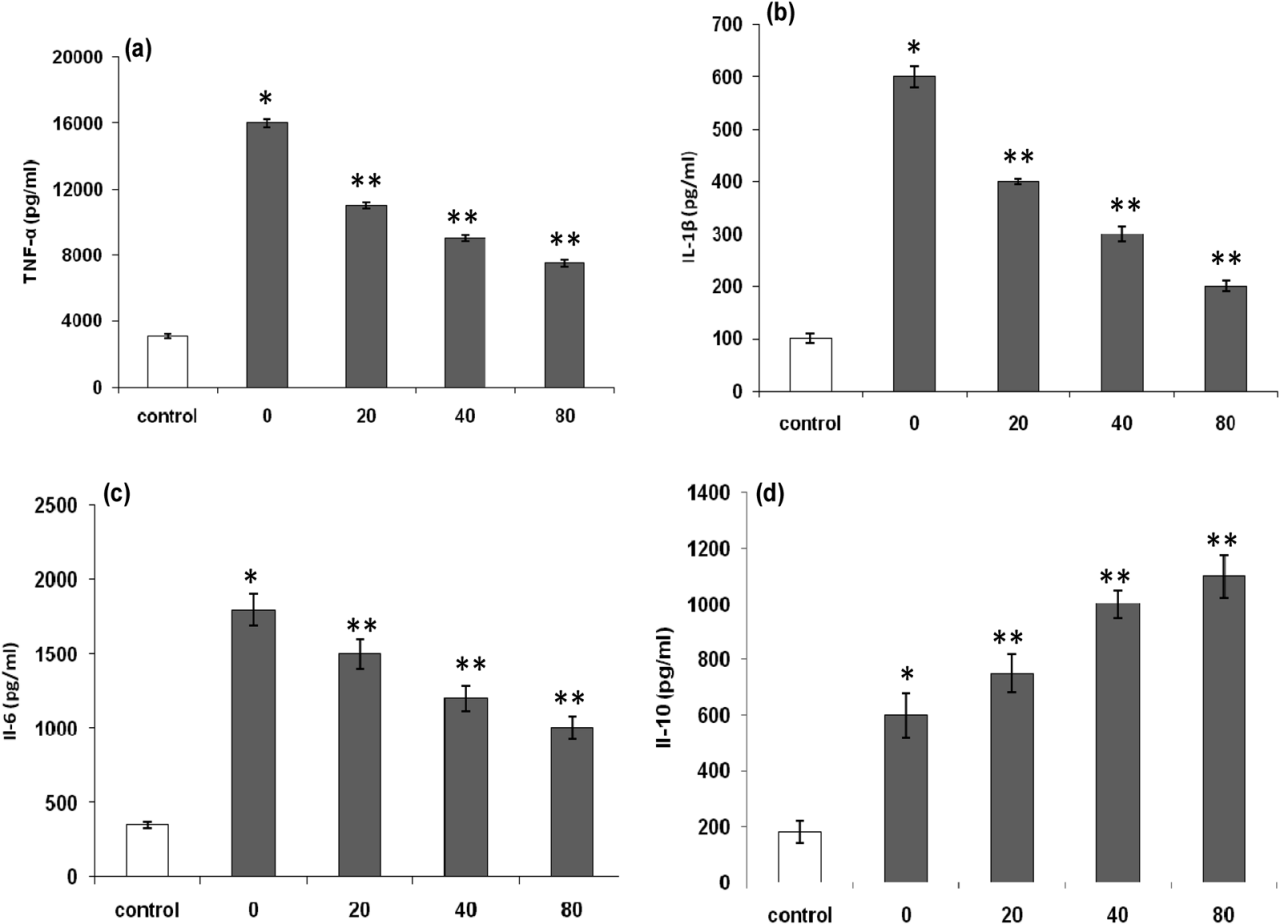
(a) Effects of *Lm*EO on lipopolysaccharide (LPS)-induced tumor necrosis factor (TNF)-α production of RAW 264.7 macrophages; (b) effects of *Lm*EO on LPS-induced interleukin (IL)-1β production of RAW 264.7 macrophages; (c) effects of *Lm*EO on LPS-induced IL-6 production of RAW 264.7 macrophages; and (d) effects of *Lm*EO on LPS-induced IL-10 production of RAW 264.7 macrophages. Values are expressed as mean ± SEM (*n* = 3). *P < 0.05 compared with the control group. **P < 0.01 compared with the LPS only treatment group.

Proinflammatory cytokines, such as TNF-α, IL-1β, IL-6, and the anti-inflammatory cytokine IL-10, play important roles in the inflammatory process. The treatment of RAW 264.7 cells with LPS alone resulted in increased release of pro and anti-inflammatory cytokines (TNF-α, IL-1β, IL-6, and IL-10) compared with non-activated controls (Fig. 7).

The increased levels of TNF-α (Fig. 7a), IL-1β (Fig. 7b), and IL-6 (Fig. 7c) in RAW 264.7 cells by LPS stimulation remarkably decreased in a dose-dependent manner after exposure of the cells to *Lm*EO (*p* < 0.05). In contrast, the levels of anti-inflammatory cytokine IL-10 significantly increased in a dose-dependent manner after the cells were exposed to *Lm*EO (*p* < 0.05; Fig. 7d).

#### Effects of *Lm*EO on LPS-induced protein expression of iNOS and COX-2

**Fig. 8:**
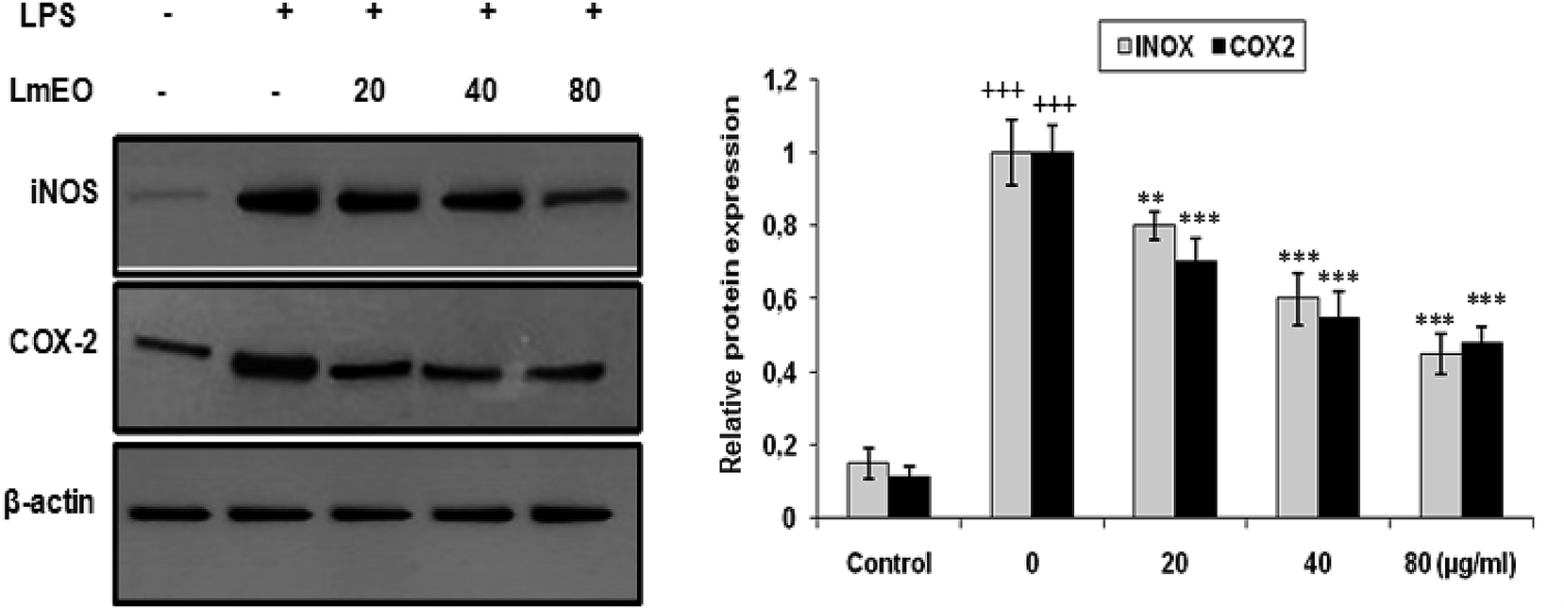
Inhibitory effects of *Lm*EO on protein expression of iNOS and COX-2 in LPS-stimulated RAW 264.7 cells. The results presented are representative of three independent experiments. Values are expressed as mean ± SEM (*n* = 3). ^+++^*P* < 0.001 compared with the control group. ** and ***P < 0.01 and ^+++^*P* < 0.001, respectively, compared with the LPS-only treatment group.

To investigate whether the inhibitory effects of *Lm*EO on the production of NO were mediated by inhibition of gene expression, we evaluated iNOS and COX-2 using western blot analysis. Macrophages were treated with *Lm*EO (20, 40 and 80 μg mL^−1^) to examine the protein expression of inflammation-associated molecules triggered by LPS (Fig. 8).

In these experiments, LPS-activated macrophages increased the protein expression levels of COX-2 and iNOS compared with those in the untreated control group. In contrast, *Lm*EO treatment down-regulated the expression of these LPS-stimulated proteins in a concentration-dependent manner (*p* < 0.05).

## Discussion

In the current study, the role of *L. maritima* leaf EO on the oxidative stress induced by CCl_4_ hepatotoxicity was investigated in male rats. The dose of CCl_4_ was selected based on the literature.^38^ The selected dose of *Lm*EO was based on the acute toxicity studies. *Lm*EO is mainly composed of oxygenated monoterpenes and monoterpene hydrocarbons in respective amounts of 74.4% and 16.5%. The major component is linalool, which accounts for 22.43% of the whole oil. To ascertain the *Lm*EO antioxidant potential, we chose the DPPH method because the stable DPPH radical is widely used to evaluate the free radical-scavenging activity of many plants. Antioxidants are able to scavenge the radical by hydrogen donation, thus inducing a decrease of the DPPH absorbance at 517 nm. The assessment of the *Lm*EO antioxidant activity shows that it is a free radical scavenger and may act as a primary antioxidant. This activity can be ascribed to the chemical composition of *Lm*EO. *Lm*EO monoterpenes may act as radical scavenging agents. It appears to be a general trend that *Lm*EO, which contains monoterpene hydrocarbons, oxygenated monoterpenes and/or sesquiterpenes, has great antioxidative properties.^39^ Monoterpene hydrocarbons, particularly terpinolene, α-terpinene and γ-terpinene, may also account for the antioxidant activity. However, this activity is less important than that of the oxygenated monoterpenes. On the other hand, low antioxidant activity is associated with sesquiterpene hydrocarbons and their oxygenated derivatives.^40^ Numerous reports clearly demonstrate the importance of medicinal plants in the treatment of oxidative stress-induced cell death.^41^ The present study was undertaken to study the possible hepatoprotective role of *L. maritima* leaf EO in a CCl_4_-induced liver toxicity rat model. High serum levels of hepatic enzymes (ALT, AST, ALP, and LDH) constitute a sensitive indicator of liver cell injury and are very helpful in recognizing hepatic diseases.^42^ Hence, free radical scavenging helps protect against CCl_4_-induced oxidative injury. In the present study, the protective effects of *Lm*EO against CCl_4_-induced hepatotoxicity and oxidative stress were investigated. According to our results, the pre-treatment with *Lm*EO attenuated the increased serum levels of liver enzymes (AST, ALT, ALP and LDH) induced by CCl_4_ toxicity with values similar to those of control rats; this was indicated by the structural and functional integrity of hepatic parenchyma cells.

In this investigation, CCl_4_ induced an increase in hepatic LPO in the experimental group II animals, expressed in terms of high TBARS level in the liver tissue of these animals. Compared to the control animals, hepatocellular damage was indicated as previously described.^43^ Pre-treatment with the *Lm*EO exhibited a significant decrease in TBARS level in the liver tissue, indicating an inhibitory role of *Lm*EO against lipid peroxidation. Consequently, we noted a decrease of CCl_4_-induced hepatic damage. The prevention of lipid peroxidation can be attributed to the ability of *Lm*EO to scavenge ROS.

ROS, such as superoxide anions and H_2_O_2_, are produced during normal aerobic metabolism, and their intracellular concentration depends on the rate of their production and their removal by various antioxidants. The antioxidant system in mammalian cells is mainly represented by three enzymes: superoxide dismutase (SOD), catalase (CAT) and glutathione peroxidase (GPx). These enzymes act in a synergic way to detoxify the superoxide anions and H_2_O_2_ in cells. In our study, the SOD, CAT and GPx activities significantly decreased in the liver tissue of CCl_4_-treated rats (group II) compared to the control group. The reduced activity of these enzymes may be due to enhanced lipid peroxidation or inactivation of the antioxidative enzymes.^44^ Intra-peritoneal injection of *Lm*EO for 14 days in group IV rats abolished the CCl_4_-induced reduction in the SOD, CAT and GPx activities, protecting liver tissue against the oxidative insults of CCl_4_. The histological data basically supported the results relative to the serum enzyme assays. We noticed massive fatty changes, such as necrosis, infiltration of lymphocytes, and several ballooning degenerations of hepatocytes, in the livers of CCl_4_-intoxicated rats; meanwhile, for rats pre-treated with *Lm*EO and subsequently given CCl_4_, the liver histopathological data revealed more or less normal architectures.

These results suggest that *Lm*EO reduces oxidative stress by preventing the generation of free radicals. The reduced oxidative stress and lipid peroxidation occurring in the *Lm*EO-treated animals can be attributed to the antioxidant potential of *Lm*EO. *Lm*EO antioxidants are able to decompose free radicals by quenching ROS and trapping radicals before they reach their cellular targets. The antioxidant activity of *Lm*EO can also be assigned to its monoterpenes. Moreover, the measured antioxidant activities may be due to the synergistic effects of two or more *Lm*EO constituents. In this context, many authors have reported that most natural antioxidative compounds act synergistically to produce a broad spectrum of antioxidative properties that create an effective defence system against free radicals.^45,46^

As natural volatile substances from plants, EOs may represent an alternative source of anti-inflammatory agents. These oils consist of mixtures containing many bioactive compounds that are biodegradable into nontoxic products and potentially suitable for use in integrated management programs.^47^ In the present study, we also investigated the anti-inflammatory effects of *Lm*EO on LPS-stimulated RAW 264.7 macrophage cells. To our knowledge, this study is the first to assess the chemical composition and bioactivity of the genus *Lobularia*.

Inflammation is a bodily response to harmful stimuli such as injury and infection.^48^ Various inflammatory models allow evaluation of test compounds and provide further understanding of the inflammatory process. In many studies, anti-inflammatory compounds have been investigated for their potential inhibitory effects *in vitro* using LPS-stimulated RAW 264.7 macrophages. LPS, a component of the outer membrane of Gram-negative bacteria, can activate murine macrophages; this induces oversecretion of various inflammatory and toxicity-mediating molecules, such as TNF-α, IL-6, eicosanoids, and NO.^49^ At concentrations of 20–80 μg mL^−1^, *Lm*EO did not show any cytotoxic effects on cells. It suppressed NO production in the LPS-treated cells in a dose-dependent manner. Ko *et al.* investigated the potential anti-inflammatory effects of *Lindera erythrocarpa* EO (LEO) through the evaluation of NO production in LPS-stimulated RAW 264 cells.^50^ They reported that NO production was substantially higher in LPS-treated cells than in untreated cells. According to the same authors, LEO suppressed NO production in LPS-treated cells in a dose-dependent manner.

In our case, LPS-activated macrophages increased the protein expression levels of COX-2 and iNOS compared with those in the untreated control group. In contrast, *Lm*EO treatment down-regulated the expression of these LPS-stimulated proteins in a concentration-dependent manner. Similar facts have been reported by Ko *et al.*, who used western blot analyses to determine whether the inhibitory activity of LEO on the production of NO and PGE2 was related to the expression levels of iNOS and COX-2.^50^ Ko *et al.* noted increased expression levels of iNOS and COX-2 in LPS-stimulated cells in comparison with untreated controls.^50^ Furthermore, they reported that LEO inhibited an LPS-induced increase in iNOS and COX-2 levels in a dose-dependent manner. These results are consistent with the inhibitory effects of LEO on the production of NO and PGE2. Additionally, EO extracted from *Hibiscus sabdariffa* was characterized by excellent anti-inflammatory activity in LPS-stimulated macrophage RAW 264.7 cells. It induced NO inhibition, which reached 67.46% at an EO concentration of 200 μg mL^−1^.^51^

To evaluate the anti-inflammatory mechanism mediated by *L. maritima*, we investigated the effects of *Lm*EO on LPS-induced cytokine production, including proinflammatory cytokines such as IL-1β and IL-6 as well as TNF-α and the anti-inflammatory cytokine IL-10, which are regarded as crucial anti-inflammatory targets.^52,53^

Proinflammatory cytokines, such as TNF-α, IL-1β, and IL-6, are primarily produced by activated monocytes or macrophages. IL-6 is characterized as an inflammatory factor because it synergistically consolidates the inflammatory actions of IL-1 in human synovial cells.^54^ According to our results, the treatment of RAW 264.7 cells with LPS alone resulted in a significant increase in proinflammatory cytokine production compared with that in the control group (*p* < 0.001). In contrast, *Lm*EO significantly reduced these cytokine levels (*p* < 0.001; Fig. 7). IL-10 is generally considered as an anti-inflammatory and immunosuppressive cytokine. Its inhibitory effects on the production of inflammatory cytokines, including TNF-α, IL-1β, and IL-6, have been reported in numerous studies.^55^ In the current study, *Lm*EO significantly increased the levels of anti-inflammatory cytokine IL-10 (Fig. 7d). These results are in concordance with those of Ko *et al.*, who reported that IL-6 and TNF-α production was considerably increased in LPS-stimulated RAW 264.7 cells but significantly inhibited by LEO in a dose-dependent manner.^50^ Similar results have been reported for curcumin. Compared to LPS-stimulated controls, curcumin led to a dose-dependent reduction in the levels of proinflammatory cytokines. LPS-stimulated production of TNF-α was reduced in a dose-dependent manner by cells exposed to curcumin. TNF-α is a major cytokine involved in inflammation.^56^ Curcumin supplementation also resulted in inhibition of LPS-induced IL-10 and IFN-γ. Moreover, curcumin downregulated IL-6 and TNF-α production.^56^ Therefore, the regulation of cytokines observed in this study may reflect one of the mechanisms underlying the anti-inflammatory effects of *Lm*EO.

## Conclusion

*In vivo* investigations were performed to explore the therapeutic role of a halophyte plant in CCl_4_-induced liver injury for the first time. The main results of the current *in vivo* study revealed that *Lm*EO has hepato-protective effects against CCl_4_-induced oxidative stress in rats, as evidenced by the decreased TBARS levels in liver tissue and the decrease of liver marker enzyme levels in serum to their normal values. Moreover, *Lm*EO exerted potent anti-inflammatory effects through down-regulating the expression of multiple inflammatory cytokines and related mediators, including IL-1, IL-6, TNF-α, NO, iNOS, and COX-2, in LPS-induced macrophages. These significant findings not only support the traditional use of some medicinal plants in the treatment of various inflammation-associated diseases, but also provide evidence that *Lm*EO can be exploited to develop new potential therapeutic agents for inflammatory diseases. As a continuation of the present study, isolation and investigation of the *Lm*EO constituents responsible for these hepatoprotective and anti-inflammatory effects should be undertaken in order to confirm and elucidate the mechanisms behind these activities.

## Conflicts of interest

The authors declare that there are no conflicts of interest.

## Supplementary Material
